# Processing of Brain Signals by Using Hemodynamic and Neuroelectromagnetic Modalities

**DOI:** 10.1155/2010/934180

**Published:** 2010-04-14

**Authors:** Laura Astolfi, Sara Gonzalez Andino, Fabrizio De Vico Fallani, Fabio Babiloni

**Affiliations:** ^1^IRCCS Fondazione Santa Lucia, Via Ardeatina 306, 00179 Roma, Italy; ^2^Department of Computer Science and System, Faculty of Engineering, Via Ariosto 25, University of Rome “Sapienza”, 00185 Rome, Italy; ^3^Electrical Neuroimaging Group, Department of Clinical Neuroscience, University of Geneva, 1211 Geneva 14, Switzerland; ^4^Department of Physiology and Pharmacology, University of Rome “Sapienza”, 00185 Rome, Italy

Human neocortical processes involve temporal and spatial scales spanning several orders of magnitude, from the rapidly shifting somatosensory processes characterized by a temporal scale of milliseconds and a spatial scales of few square millimeters to the memory processes, involving time periods of seconds and spatial scale of square centimeters. Information about the brain activity can be obtained by measuring different physical variables arising from the brain processes, such as the increase in consumption of oxygen by the neural tissues or a variation of the electric potential over the scalp surface. All these variables are connected in direct or indirect way to the neural ongoing processes, and each variable has its own spatial and temporal resolution. The different neuroimaging techniques are then confined to the spatiotemporal resolution offered by the monitored variables. For instance, it is known from physiology that the temporal resolution of the hemodynamic deoxyhemoglobin increase/decrease lies in the range of 1-2 seconds, while its spatial resolution is generally observable with the current imaging techniques at few millimeter scale. Today, no neuroimaging method allows a spatial resolution on a millimeter scale and a temporal resolution on a millisecond scale. Nevertheless, the issue of several temporal and spatial domains is critical in the study of the brain functions, since different properties could become observable, depending on the spatiotemporal scales at which the brain processes are measured. 

It is well knownm that the electroencephalography (EEG) and magnetoencephalography (MEG) are useful techniques for the study of brain dynamics, due to their high temporal resolution. However, it has been said that the spatial resolution of the EEG is rather low, due to the different electrical conductivities of brain, skull, and scalp that markedly blur the EEG potential distributions, making the localization of the underlying cortical generators problematic. In the last ten years, a body of mathematical techniques, known as high-resolution EEG, was developed to estimate precisely the cortical activity from noninvasive EEG measurements. Such techniques include the use of a large number of scalp electrodes, realistic models of the head derived from magnetic resonance images (MRIs), and advanced processing methodologies related to the solution of the so-called “inverse problem,” that is, the estimation of the brain activity (i.e., electromagnetic generators) from the EEG/MEG measurements. The approach implies both the use of thousands of equivalent current dipoles as a source model and the realistic head models, reconstructed from magnetic resonance images, as the volume conductor medium. The use of geometrical constraints on the position of the neural source or sources within the head model generally reduces the solution space (i.e., the set of all possible combinations of the cortical dipoles strengths). An additional constraint is to force the dipoles to explain the recorded data with a minimum or a low amount of energy (minimum-norm solutions). The solution space can be further reduced by using information deriving from hemodynamic measures (i.e., fMRI-BOLD phenomena) recorded during the same task. The rationale of a multimodal approach is that neural activity, modulating neuronal firing and generating EEG/MEG potentials, increases glucose and oxygen demands. This results in an increase in the local hemodynamic response that can be measured by functional magnetic resonance images (fMRIs). Hence, fMRI responses and cortical sources of EEG/MEG data can be spatially related, and the fMRI information can be used as a prior in the solution of the inverse problem. As a result of all these computational approaches, it is possible to estimate the cortical activity with a spatial resolution of few millimeters and with a temporal resolution of milliseconds from noninvasive EEG measurements. 

In the framework of a COST Action BM0601 NeuroMath, there was organized a workshop in Rome in 2009 on the themes of the processing of neuroelectromagnetic and hemodynamic signals. Selected papers from this conference were subjected to standard peer-review and compiled in this special issue. With this issue we want to illustrate ongoing and emerging research in the development and application of mathematical methods to the recording, analysis, integration, and modeling of neural activity. The selected papers, written by world class scientists, cover diverse issues ranging from computational models to concrete applications of the methods within the neurosciences. We hope that the readership of CIN could appreciate this special issue as we appreciated it during its composition.

*Laura Astolfi* *Sara Gonzalez Andino* *Fabrizio De Vico Fallani* *Fabio Babiloni*

